# Genotoxicity evaluation of So-ochim-tang-gamibang (SOCG), a herbal medicine

**DOI:** 10.1186/s12906-018-2111-2

**Published:** 2018-02-02

**Authors:** Mi Young Lee, Yang-Chun Park, Mirim Jin, Eunseok Kim, Jeong June Choi, In Chul Jung

**Affiliations:** 1 Genogen Co., Ltd, Room 402, 125, Osongsaengmyeong 2-ro, Osong-eup, Heungdeok-gu, Cheongju-si, Chungcheongbuk-do 28161 Republic of Korea; 2 Department of Internal Medicine, College of Korean Medicine, Daejeon University, 62 Daehak-ro, Dong-gu, Daejeon, 34520 Republic of Korea; 3 College of Medicine, Gachon University, Incheon, 23051 Republic of Korea; 4 Department of Acupuncture & Moxibustion Medicine, College of Korean Medicine, Kyung Hee University, Seoul, 02447 Republic of Korea; 5 Laboratory of Molecular Medicine, College of Korean Medicine, Daejeon University, 62 Daehak-ro, Dong-gu, Daejeon, 34520 Republic of Korea; 6 Department of Neuropsychiatry, College of Korean Medicine, Daejeon University, 62 Daehak-ro, Dong-gu, Daejeon, 34520 Republic of Korea

**Keywords:** SOCG, So-ochim-tang-gamibang, Genotoxicity, Bacterial reverse mutation test, Chromosomal aberration test, Micronucleus test, Korean medicine

## Abstract

**Background:**

So-ochim-tang-gamibang (SOCG) is a traditional Korean medicine frequently used for depression in the clinical field. In this study, we evaluated the potential genotoxicity of SOCG using three standard batteries of tests as part of a safety evaluation.

**Methods:**

SOCG was evaluated for potential genotoxic effects using the standard three tests recommended by the Ministry of Food and Drug Safety (MFDS) of Korea. These tests were the bacterial reverse mutation test (Ames test), in vitro mammalian chromosomal aberration test using Chinese hamster lung cells, and in vivo micronucleus test using ICR mice.

**Results:**

The Ames test with *Salmonella typhimurium* strains TA98, TA100, TA1535 and TA1537 and the *Escherichia coli* strain WP2uvrA(pKM101) showed that SOCG did not induce gene mutations at any dose level in all of the strains. SOCG did not induce any chromosomal aberrations in the in vitro chromosomal aberration test (for both the 6 and 24 h test) and the in vivo micronucleus test.

**Conclusions:**

Based on the results of these tests, it was concluded that SOCG does not exhibit any genotoxic risk under the experimental conditions of this study.

## Background

Korean medicine has a long history with numerous clinical experiences. Herbal medicine is one of the main treatments in Korean medicine. There are several Korean herbal medicines prepared from a single herb. However, the composition of several kinds of medicinal herbs are widely used. Long-time experiences in clinical practice has led the practitioners of Korean medicine to understand the toxicity of each medicinal herb and the necessary precautions in practice. Moreover, it is known in Korean medicine that the combination of each medicinal herb strengthens or neutralizes the main efficacy and sometimes generates toxicity [1]. A historic understanding of medicinal herbs has led people to think that natural treatments are safer than that of other medications; as a result, there are insufficient toxicological scientific data on Korean medicine.

The Food and Drug Administration of Korea enacted a safety guideline (MFDS guideline Notification No. 2013–121) through the Ministry of Food and Drug Safety for new drugs [2]. This guideline suggested by the MFDS evaluates the genotoxicity of new or modified natural medicine formulae. The guideline stipulates that Korean medicine is also subjected to the toxicity evaluation. Genotoxicity assays tests the toxicity potential of a drug to induce genetic modifications [3].

SOCG is a Korean medicine herbal formula, which consists of 6 medicinal herbs as follows: Cyperi Rhizoma (*Cyperus rotundus* L.), Lindera Radix (*Lindera strychnifolia* Fern.-Vill.), Aucklandiae Radix (*Auckladia lappa* Decne.), Glycyrrhizae Radix (*Glycyrrhiza uralensis* Fisch.), Aurantii Fructus (*Citrus aurantium* L.), and Platycodi Radix (*Platycodon grandiflorus* Jacq. A.DC). SOCG has been used in clinical practice to ameliorate stress-induced depressive disorders and has been reported to suppress depressive- and anxiety-like animal behaviors by protecting against neuronal cell death [4]. SOCG was also suggested to modulate the production of serotonin expression accompanied by decreased mRNA levels of 5-hydroxytryptamine transporter and Tryptophan hydroxylase 1. Additionally, the original formula, Soochim-tang, has been used to treat depressive moods and somatoform pain induced by psychiatric disorders [5]. In previous study, *C. rotundus* extracts did not show genotoxicity in Balb/c mice up to 300 mg/kg [6]. Although the genotoxicity of *C. aurantium* is not well known, it is supposed to do not have the toxicity. Because the methanol extract of the herb had reported that it could suppress the furylfuramide-induced mutagenesis by suppressing *umu* gene expression in *Salmonella typhimurium* TA100 [7]. However, the genotoxic potential of the SOCG extract has not been investigated thus far.

In this study, we investigated the potential genotoxicity of SOCG using the standard tests, the bacterial reverse mutation test (Ames test), in vitro mammalian chromosomal aberration test, and in vivo micronucleus test, which are recommended by the Ministry of Food and Drug Safety (MFDS) of Korea (MFDS guideline Notification No. 2013–121).

## Methods

### Preparation of the SOCG

The SOCG was prepared from six crude herbs, Cyperi Rhizoma (*Cyperus rotundus* L.), Lindera Radix (*Lindera strychnifolia* Fern.-Vill.), Aucklandiae Radix (*Auckladia lappa* Decne.), Glycyrrhizae Radix (*Glycyrrhiza uralensis* Fisch.), Aurantii Fructus (*Citrus aurantium* L.) and Platycodi Radix (*Platycodon grandiflorus* Jacq. A.DC), which were purchased from Dong Kyung Pharmaceutical (Seoul, Korea). The SOCG was formulated by boiling the six herbs (ratio 8: 4: 1: 1: 4: 4, in the order given above. This ratio is the standard formula in clinical application.) in distilled water at 100 °C for 2 h. The volume of distilled water was 10 times the total herb weight (110 kg/1100 L, *w*/*v*). This decoction was evaporated under reduced pressure at 60 °C for 5 h and freeze-dried. The extracted SOCG powder was stored at − 20 °C. Voucher specimens (No. 194A079–85) of the collected herb samples were deposited in the herbarium of Han Kook Shin Yak (Nonsan, Korea). The SOCG batch used in this study was identical with the batch used in a previous study [4]. The SOCG powder was reconstituted in distilled water (Choongwae Pharma, Korea) just before use.

### Bacterial strains

*Salmonella typhimurium* strains TA98, TA100, TA1535 and TA1537 and *Escherichia coli* strain WP2*uvrA*(pKM101) were used as the tester strains. The TA98 and TA1537 strains were used to detect frame-shift mutagens and TA100, TA1537 and WP2*uvrA*(pKM101) strains were used for detecting base-pair substitution mutagens. These strains were purchased from Molecular Toxicology, Inc. (MOLTOX™, U.S.A.) on October 28, 2011.

### Cell line

The Chinese Hamster Lung cell line was purchased from the American Type Culture Collection (ATCC, U.S.A.) on Nov. 24, 2011. The modal chromosome number of this cell line is 25. The doubling time is approximately 15 h. Cells were cultured in Eagle’s Minimum Essential Medium (EMEM, Lonza Walkersville, U.S.A.) supplemented with 10% fetal bovine serum (FBS, Gibco, U.S.A.) and incubated in a 5% CO_2_ incubator (MCO-20AIC, SANYO, Japan) at 37 °C. The cell line was evaluated for contamination of mycoplasma with the Hoechst Stain Kit (MPBIOMEDICALS, Japan). Sub-culturing was done approximately twice a week.

### Animals and husbandry

Specific pathogen-free ICR male mice (28.3–32.7 g) 7 weeks old were purchased from Orientbio and used after one week of quarantine and acclimatization. This study was reviewed and approved by the Institutional Animal Care and Use Committees (IACUC) of Biotoxtech Co., Ltd. based on the Animal Protection Act (Enactment May 31, 1991, No. 4379, Revision Aug. 13, 2013, No. 12051). The animals were housed in a good laboratory practice (GLP) facility with controlled temperature (20.4–23.4 °C), humidity (44.1–69.1%), ventilation (10–15 times per hour), light (12:12 h light:dark cycle), and illumination (150–300 Lux). Feed (Teklad Certified Irradiated Global 18% Protein Rodent Diet 2918C, Harlan Laboratories, U.S.A.) and sterilized public tap water filtered and irradiated by ultraviolet light were provided ad libitum*.*

### S9 mix

S9 consisting of phenobarbital and 5,6-benzoflavone-induced S9 fraction and cofactor were purchased from Oriental Yeast in Japan. The S9 mix was used as a metabolic activation system. The cofactor for the Ames test contained 0.4 mol/L MgCl_2_, 1.65 mol/L KCl, 1.0 mol/L glucose-6-phosphate, 0.1 mol/L NADPH, 0.1 mo/L NADH, 0.2 mol/L sodium phosphate buffer (pH 7.4) and purified water. The cofactor for the chromosomal aberration test contained 50 mmol/L MgCl_2_, 330 mmol/L KCl, 50 mmol/L glucose-6-phosphate, 40 mmol/L NADP, 20 mmol/L HEPES buffer (pH 7.2) and purified water. The protein content of S9 was 20.3–23.2 mg/mL S9, and the cytochrome P450 content was 2.40–2.86 nmol/mg microsomal protein. In the Ames test, the final concentration of S9 in the S9 mix was 10% *v*/v. In the chromosomal aberration test, the final concentration of S9 in the culture medium was approximately 5% v/v. Fresh S9 mix was prepared before use.

### Bacterial reverse mutation test (Ames test)

The Ames test was conducted in accordance with the MFDS guideline (Notification No.2013–121). Histidine auxotroph mutants of *Salmonella typhimurium* strains TA98, TA100, TA1535 and TA1537 and a tryptophan auxotroph mutant of *Escherichia coli* strain WP2*uvrA*(pKM101) were cultured in nutrient broth No.2 medium for 8.5 h in a shaking water bath (37 °C, 130 rpm). The bacteria strains are widely used for Ames test due to the high sensitivity to mutagens. MFDS, OECD, and ICH guidelines suggest the strains for bacterial reverse mutation test [2, 8, 9]. The turbidity of the cultures was measured with a UV/VIS spectrophotometer (660 nm, V-550, Jasco, Japan). Cultures with a density greater than 1 × 10^9^ cells/mL were used in this test. Positive controls used in this test were 2-Nitrofluorene (2-NF, Sigma-Aldrich, U.S.A.), Sodium azide (SA, Sigma-Aldrich, U.S.A.), 9-Aminoacridine (9-AA, Sigma-Aldrich, U.S.A.), 2-(2-furyl)-3-(5-nitro-2-furyl)acrylamide (AF2, Wako, Japan) and 2-Aminoanthracene (2-AA, Sigma-Aldrich, U.S.A.); the negative control was water for injection (Choongwae Pharma, Korea).

In the presence of metabolic activation, 100 μL of each the SOCG, strain-specific positive control, or negative control were mixed with 500 μL of S9 mix and 100 μL of pre-incubated bacterial suspension. These mixtures were incubated in a shaking water bath at 37 °C for 20 min. Then, 2 mL of warmed top agar for *Salmonella typhimurium* were added to the TA98, TA100, TA1535 and TA1537 strains, and 2 mL of top agar for *Escherichia coli* were added to the WP2*uvrA*(pKM101) strain. Finally, these mixtures were poured onto minimal glucose agar plates. In the absence of metabolic activation, 500 μL of 0.1 mol/L sodium phosphate buffer (pH 7.4) instead of S9 mix were added, and the rest of procedure was carried out with the same method as above. After the top agar was solidified, the plates were cultured in an incubator at 37 °C for 48 h. A preliminary dose range-finding study showed that SOCG did not have growth inhibition to 5000 μg/plate, which is the highest dose level for the Ames test. Triplicate plates were used per dose in the test. The result of the Ames test was considered to be positive when the number of revertant colonies for any strain at one or more doses was increased at least two times when compared to the negative control value and there was a dose dependency or reproducibility as the dose increased. In addition, the growth inhibition was terminated when there was a clearing or diminution of the background lawn, the appearance of micro-colonies or a decrease of more than 50% in the number of revertant colonies compared to the negative control.

### In vitro mammalian chromosomal aberration test

The chromosomal aberration test was conducted in accordance with the MFDS guideline (Notification No.2013–121). We used Chinese hamster lung cells for our study. This cell line is suggested by MFDS, OECD, and ICH guidelines, and widely used for in vitro mammalian chromosomal aberration test due to the high sensitivity [2, 9–11]. The experimental protocol was performed with three treatment systems: short duration treatments with and without metabolic activation and continuous treatment without metabolic activation. A preliminary cell growth inhibition study was done to determine the highest dose levels for the chromosomal aberration test [12] and 5000 μg/mL was set the highest dose for all the systems. The calculated 50% growth inhibitory concentrations (IC_50_) were approximately 3438.1 μg/mL and 2287.5 μg/mL for the short duration treatments with and without metabolic activation, respectively, and 709.0 μg/mL for the continuous treatment without metabolic activation. Based on the results, the highest dose levels were as 3500 μg/mL, 2300 μg/mL, and 710 μg/mL for each system. Positive controls used in this test were mitomycin C (Sigma-Aldrich, U.S.A.) and benzo[α]pyrene (Sigma-Aldrich, U.S.A.); the negative control was distilled water for injection (Choongwae Pharma., Korea). Each treatment was performed in duplicate. For the short duration treatments, the cells were treated with the SOCG for 6 h, and cell culture medium was exchanged to fresh. The cells were cultured for an additionally 18 h. For the continuous treatment, the cells were treated with the test substance for 24 h. Chromosome slides were prepared for a 1.5 cell cycle length after the start of treatment. Two hours prior to the culture completion, colcemid (0.2 μg/mL, Gibco, U.S.A.) was added. Following culture completion, the collected cells were treated with 0.075 mol/L KCl solution (prewarmed at 37 °C) at 37 °C for 20 min and fixed with an ice-cold fixative mixture (methanol:acetic acid = 3:1). The cells were dropped onto clean dry slides and stained with a 3% Giemsa solution for 20 min. Two hundred metaphases per dose were observed with a light microscope (× 400–600 magnification, BX51, Olympus, Japan). The chromosomal aberration was classified into structural aberration, numerical aberration and other. Structural aberrations that were recorded included chromatid break (ctb), chromatid exchange (cte), chromosome break (csb), chromosome exchange (cse), gap and fragmentation (frg). Numerical aberrations that were recorded included polyploidy (pol) and endoreduplication (end). For the above aberrations, any cell with one or more aberrations was counted as one aberrant cell. For gap, the number of cells excluding gap was recorded and calculated. The identification of a chromosome aberration was carried out according to JEMS-MMS [13]. The result of the in vitro mammalian chromosomal aberration test was evaluated in accordance with the criteria of Toshio Sofuni [14]. For the positive controls, the frequency of cells with aberrations was above 10%; for equivocal, it was above 5% and below 10%, and for negative, it was below 5%.

### In vivo micronucleus test

The micronucleus test was conducted in accordance with the MFDS guideline (Notification No.2013–121). SOCG was administered once by gavage to male ICR mice at doses of 1250, 2500, and 5000 mg/kg with 10 mL/kg volume. Mice in the negative control group received only the vehicle (water for injection; Choongwae Pharma, Korea) by oral gavage. Mitomycin C (Sigma-Aldrich, U.S.A.) was administered by intraperitoneal injection at a dose of 2 mg/kg as a positive control. The animals were randomly assigned to 5 groups (*N* = 5/each group). All animals were observed daily for clinical signs, and individual body weights were recorded just prior to administration and prior to harvesting the bone marrows cells. The bone marrow cells were harvested at 24 h after administration. Immediately following sacrifice by cervical dislocation, femurs were dissected from each animal and trimmed. Bone marrow cells were collected by rinsing the canal with fetal bovine serum 200 μL and centrifuged at 1000 rpm for 5 min (4 °C). The precipitate was mixed well and placed onto a clean dry slide and spread with 1 drop of cell suspension. The slides were air-dried, fixed with methanol for 5 min, and stained with a 3% Giemsa staining solution (0.01 mol/L Sörenson phosphate buffer solution, pH 6.8) for 30 min. The stained slides were washed with 0.01 mol/L Sörenson phosphate buffer solution (pH 6.8) and 0.004% citric acid solution. Slides were evaluated under a microscope (BX51, Olympus, Japan) at 600–1000-fold magnification. A total of 2000 polychromatic erythrocytes (PCE) per animal were scored for determining the frequency of micronucleus polychromatic erythrocytes (MNPCEs). To evaluate the myelotoxic effects, 500 erythrocytes were scored per animal to determine the ratio of PCE to the total number of erythrocytes. The result of the in vivo micronucleus test was considered to be positive when the frequency of the MNPCEs was statistically significantly increased according to the criteria of Kastenbaum & Bowman [15].

### Statistical analysis

The statistical analysis was done with the SAS program (version 9.3, SAS Institute, U.S.A.). For the aberration cell data from the in vitro chromosomal aberration test, Fisher’s exact test was used for the comparison of the negative control group with the test substance groups or the positive control group (significance levels: 0.05 and 0.01, two-tailed). The ratio of PCE among total erythrocytes and body weight data were analyzed with Bartlett’s test for homogeneity of variance (significance level: 0.05). Data showing homogeneous variance were analyzed with one-way analysis of variance (ANOVA) for homogeneous data (significance level: 0.05).

## Results

### Bacterial reverse mutation test

The result of the bacterial reverse mutation test is shown in Table 1. The mean number of revertant colonies from the test substance was less than twice that of the negative control value in the presence and absence of metabolic activation at any dose level in all the strains. In the positive control group, the mean number of revertant colonies was markedly increased when compared to the negative control value. Growth inhibition by the test substance and precipitation were not observed at any dose level in all the strains. The number of revertant colonies in the negative and positive control groups was within the range of the historical control data.

**Table 1:** Results of the bacterial reverse mutation test for SOCG

	TA98	TA100	TA1535	TA1537	WP2*uvrA* (pKM101)
Dose (μg/plate)	Number of revertant colonies/plate (in the absence of metabolic activation)^d^ [factor]^e^				
Negative control^a^	19 ± 3	52 ± 4	6 ± 2	5 ± 2	86 ± 4
313	20 ± 1[1.05]	56 ± 13[1.08]	7 ± 2[1.17]	4 ± 2[0.80]	90 ± 12[1.05]
625	19 ± 4[1.00]	50 ± 7[0.96]	6 ± 2[1.00]	6 ± 3[1.20]	92 ± 6[1.07]
1250	19 ± 4[1.00]	49 ± 7[0.94]	6 ± 2[1.00]	6 ± 1[1.20]	100 ± 5[1.16]
2500	20 ± 3[1.05]	41 ± 8[0.79]	6 ± 1[1.00]	5 ± 1[1.00]	103 ± 6[1.20]
5000	19 ± 3[1.00]	28 ± 3[0.54]	6 ± 1[1.00]	4 ± 1[0.80]	107 ± 11[1.24]
Positive control^b^	475 ± 80[25.0]	549 ± 29[10.6]	461 ± 28[76.8]	430 ± 160[86.0]	1056 ± 117[12.3]
Dose (μg/plate)	Number of revertant colonies/plate (in the presence of metabolic activation)^d^ [factor]^e^				
Negative control^a^	22 ± 3	79 ± 6	9 ± 2	13 ± 2	114 ± 15
313	21 ± 3[0.95]	73 ± 7[0.92]	10 ± 3[1.11]	14 ± 3[1.08]	113 ± 8[0.99]
625	23 ± 3[1.05]	76 ± 4[0.96]	10 ± 2[1.11]	17 ± 3[1.31]	127 ± 17[1.11]
1250	22 ± 2[1.00]	76 ± 10[0.96]	9 ± 2[1.00]	13 ± 4[1.00]	141 ± 7[1.24]
2500	21 ± 5[0.95]	72 ± 10[0.91]	11 ± 4[1.22]	15 ± 3[1.15]	147 ± 9[12.9]
5000	20 ± 6[0.91]	75 ± 4[0.95]	10 ± 3[1.11]	13 ± 1[1.00]	146 ± 6[1.28]
Positive control^c^	221 ± 10[10.0]	404 ± 4[5.11]	121 ± 18[13.4]	134 ± 9[10.3]	460 ± 28[4.04]

^a^Water for injection

^b^TA98: 2-Nitrofluorene (2-MF), 5.0 μg/plate; TA100: Sodium azide (SA), 1.5 μg/plate; TA1535: SA, 1.5 μg/plate; TA1537: 9-Aminoacridine (9-AA), 80 μg/plate; WP2*uvrA*(pKM101): 2-(2-furyl)-3-(5-nitro-2-furyl) acrylamide(AF2), 0.005 μg/plate

^c^TA98: 2-Aminoanthracene (2-AA), 1.0 μg/plate; TA100: 2-AA, 2.0 μg/plate; TA1535: 2-AA, 3.0 μg/plate; TA1537: 2-AA, 3.0 μg/plate; WP2uvrA(pKM101): 2-AA, 2.0 μg/plate

^d^Data expressed as mean ± standard deviation

^e^No. of revertant colonies per treated plate/No. of revertant colonies per negative control plate

### In vitro mammalian chromosomal aberration test

**Fig. 1:**
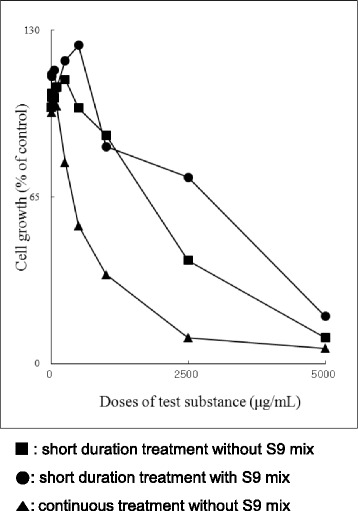
Cell growth inhibition by SOCG. Chines hamster lung cells were exposed to various doses of SOCG for the short duration treatment (6 h) without and with S9 mix or continuous treatment (24 h) without S9 mix. SOCG shows a cell dose-dependent growth inhibition

In the preliminary cell growth inhibition study, greater than 50% cytotoxicity was observed in the short duration treatments with and without metabolic activation and the continuous treatment without metabolic activation (Fig. 1). The dose levels of the in vitro mammalian chromosomal aberration test were chosen by considering the cytotoxicity of the SOCG. The result is shown in Table 2. In the slide preparation, 200 metaphase cells were not evident at high dose levels in the short duration treatments with and without metabolic activation. Therefore, three dose levels excluding the high dose were selected for the observation of chromosome aberrations. In the continuous treatment without metabolic activation, 200 metaphase cells were evident at high dose levels. Therefore, three dose levels including the high dose were selected for the observation of chromosome aberrations. The frequency of cells with chromosome aberrations was less than 5% for the short duration treatments with and without metabolic activation and for the continuous treatment without metabolic activation. There was no statistically significant difference in the frequency of cells with chromosome aberrations at any dose level of the test substance compared to the negative control group. In the positive control group, the frequency of cells with structural chromosomal aberrations was statistically significantly increased compared to the negative control group (*p* < 0.01, Fisher’s exact test). The frequency of cells with chromosome aberrations for the positive and negative controls was within the range of the historical control data.

**Table 2:** Results of the in vitro chromosomal aberration test for SOCG

Dose (μg/mL)	S9 mix	Trt-Rec Time^d^ (hrs)	Proliferation rate (%)	Structural aberrations (%)^e^					Numerical aberration (%)^g^		
				ctb	cte	csb	cse	total	end	pol	total
Short duration treatment without metabolic activation											
Negative control^a^	–	6–18	100	0.0	0.0	0.0	0.0	0.0	0.0	0.0	0.0
288	–	6–18	85.3	0.0	0.0	0.0	0.0	0.0	0.0	0.5	0.0
575	–	6–18	88.1	0.0	0.0	0.0	0.0	0.0	0.0	0.0	0.0
1150	–	6–18	76.3	0.0	0.0	0.0	0.0	0.0	0.0	0.0	0.0
2300	–	6–18	29.9	Toxic							
Positive control^b^	–	6–18		7.0	14.0	0.0	0.0	17.5*	0.0	0.0	0.0
Short duration treatment with metabolic activation											
Negative control^a^	+	6–18	100	0.5	0.0	0.0	0.0	0.5	0.0	0.0	0.0
438	+	6–18	87.8	0.0	0.0	0.0	0.0	0.0	0.0	0.5	0.5
875	+	6–18	87.8	0.0	0.0	0.0	0.0	0.0	0.0	0.0	0.0
1750	+	6–18	70.7	0.0	0.0	0.0	0.0	0.0	0.0	0.0	0.0
3500	+	6–18	57.7	Toxic							
Positive control^c^	+	6–18		7.5	0.0	0.0	19.0	23.0*	0.0	0.5	0.5
Continuous treatment without metabolic activation											
Negative control^a^	–	24–0	100	0.5	0.0	0.0	0.0	0.5	0.0	0.0	0.0
88.8	–	24–0	113	not observed							
178	–	24–0	76.2	0.0	0.0	0.0	0.0	0.0	0.0	0.0	0.0
355	–	24–0	71.4	0.0	0.0	0.0	0.0	0.0	0.0	0.0	0.0
710	–	24–0	58.7	0.0	0.5	0.0	0.0	0.5	0.0	0.0	0.0
Positive control^b^	–	24–0		5.0	26.5	0.0	0.0	30.0*	0.0	0.0	0.0

^a^Water for injection

^b^Mitomycin C, 0.1 μg/mL, ^c^ Benzo[α]pyrene, 20 μg/mL, ^d^ Trt-Rec Time: Treatment-Recovery times

^e^Structural aberration (%): The number of cells with structural chromosome aberration in metaphase cells. Ctb: chromatid breakage; cte: chromatid exchange; csb: chromosome breakage; cse: chromosome exchange; total: total number of cells with structural chromosome aberration except for gap in metaphase cells

^g^Numerical aberration (%): The number of cells with numerical chromosome aberrations in metaphase cells; end: endoreduplication; pol: polyploidy; total: total number of cells with numerical chromosome aberrations in metaphase cells

Significant difference from negative control by fisher’s exact test: * *p* < 0.01

### In vivo micronucleus test

In the preliminary dose range finding study, no signs of systemic toxicity were recorded in SOCG-treated mice. The sampling time of the bone marrow cells was chosen based on a preliminary study (data not shown). The result of the in vivo micronucleus test is shown in Table 3. There was no significant increase in the incidence of MNPCEs in the SOCG-treated groups when compared to the negative control group. There was no significant difference in the ratio of PCE among the total erythrocytes in any of the SOCG-treated groups when compared to the negative control group. The induction of MNPCEs in the negative and positive control groups was within the range of the historical control data.

**Table 3:** Results of the in vivo micronucleus test for SOCG

Dose (mg/kg)	Sampling time (hrs)	Number of MNPCE per animal					MNPCE/PCE (%) (Mean ± SD)	PCE/(PCE + NCE) (%) (Mean ± SD)
		*M*_*1*_	*M*_*2*_	*M*_*3*_	*M*_*4*_	*M*_*5*_		
0 (Water for injection)	24	0	0	0	1	2	0.030 ± 0.045	31.1 ± 4.13
1250	24	0	0	0	3	1	0.040 ± 0.065	30.6 ± 4.94
2500	24	0	0	3	3	2	0.080 ± 0.076	29.2 ± 5.78
5000	24	1	1	1	2	2	0.070 ± 0.027	30.2 ± 2.78
2 (Mitomycin C)	24	172	138	122	174	123	7.290^*^ ± 1.282	25.4 ± 4.07

Significant difference from the negative control by Kastenbaum&Bowman’s criteria: * *p* < 0.01

*Mn*: Male animal, *n* is identification number

*MNPCE*: micronucleus polychromatic erythrocytes, *PCE*: polychromatic erythrocytes, *NCE*: normochromatic erythrocyte

## Discussion

The bacterial reverse mutation test is used to detect point mutations involving substitution, addition or deletion of one or more DNA base pairs [12, 16, 17]. It is commonly used as an initial screening test because it is rapid, inexpensive and relatively easy to perform. Many carcinogens have been shown to have a high predictive value in rodents when a positive result is obtained [18]. Results from the Ames test showed that SOCG did not produce any increase in the number of revertant colonies when compared to the negative control values obtained for the tester strains TA98, TA100, TA1535, TA1537 and WP2*uvrA*(pKM101), in the presence and absence of metabolic activation. The number of revertant colonies in the negative and positive control groups was within the range of the historical control data, and the number of revertant colonies in the positive control groups was increased more than twice when compared to the negative control value. Therefore, under the conditions of this test, SOCG was not mutagenic in the bacterial tester strains used in this study up to a high dose (5000 μg/plate).

The in vitro mammalian chromosomal aberration test is used to identify agents that induce structural chromosomal aberrations in cultured chinese hamster lung (CHL) cells [19]. Chromosome mutations are the cause of many human genetic diseases, and chromosome mutations and related mechanisms causing alterations in the tumor suppressor genes and oncogenes of somatic cells are concomitant in cancer development in humans. SOCG showed a cytotoxic effect in CHL cells, and the dose levels for the chromosomal aberration test were chosen based on the 50% growth inhibitory concentration. In this test, the frequency of cells with chromosome aberrations was less than 5% for the short duration treatments with and without metabolic activation and for the continuous treatment without metabolic activation. There was no statistically significant difference in the frequency of cells with chromosome aberrations at any dose level of the test substance compared to the negative control group. In the positive control group, the frequency of cells with structural chromosomal aberrations was statistically significantly increased compared to the negative control group. The frequency of cells with chromosome aberrations for the positive and negative controls was within the range of the historical control data. Therefore, under the conditions of this test, SOCG did not induce chromosomal aberrations in CHL cells.

The in vivo micronucleus test is used to identify agents that induce chromosome damage. Micronuclei were first used to quantify chromosomal damage [20] and are now recognized as one of the most successful and reliable assays for genotoxic carcinogens [21]. This test measures clastogenicity (chromosome breakage) and aneugenicity (chromosome lagging), and estimating the ratio of PCE among the total erythrocytes is useful for evaluating any perturbations in hematopoiesis [22]. The clastogenicity of SOCG was evaluated in ICR mice. In the preliminary dose range finding study, clinical signs of toxicity and mortality were not noted in the mice treated with SOCG up to a high dose (5000 mg/kg). In this study, the incidence of MNPCE was not significantly increased when compared to the negative control group. The ratio of PCE among the total erythrocytes for the test substance groups was not significantly different when compared to the negative control group. In the positive control group, the incidence of MNPCE in PCE was significantly increased when compared to the negative control group. The incidence of MNPCE and the ratio of PCE among total erythrocytes in the positive and negative control groups were within the range of historical control data. Therefore, this test indicated that SOCG extract did not induce micronuclei in ICR mouse bone marrow cells under the conditions of this test.

## Conclusions

By using the three standard battery systems for genetic damage, it was found that SOCG is not mutagenic in the in vitro system nor clastogenic in the in vivo system. Based on these results, it is concluded that its use in herbal medicines poses no genotoxic risks.

## Abbreviations

CHL: Chinese hamster lung
D-PBS: Dulbecco’s phosphate-buffered saline
FBS: Fetal bovine serum
IACUC: The Institutional Animal Care and Use Committees
MDFS: Ministry of Food and Drug Safety
MNPCE: Micronucleus polychromatic erythrocytes
PCE: Polychromatic erythrocytes
SOCG: So-ochim-tang-gamibang
